# A Case Report of an Immature Pituitary-Specific Transcription Factor 1 (PIT1)-Lineage Pituitary Neuroendocrine Tumor Along With Its Cytology and Ultrastructural Studies

**DOI:** 10.7759/cureus.75757

**Published:** 2024-12-15

**Authors:** Nobuyuki Oguri, Takako Tokumitsu, Takashi Watanabe, Hironobu Okuyama, Yuichiro Sato

**Affiliations:** 1 Department of Pathology, Faculty of Medicine, University of Miyazaki, Miyazaki, JPN; 2 Department of Clinical Neuroscience, Section of Neurosurgery, Faculty of Medicine, University of Miyazaki, Miyazaki, JPN; 3 Department of Pathology, Section of Oncopathology and Morphological Pathology, Faculty of Medicine, University of Miyazaki, Miyazaki, JPN

**Keywords:** cytology, immature pit1-lineage tumor, pituitary, pseudonuclear inclusion, ultrastructural study

## Abstract

Immature pituitary-specific transcription factor 1 (PIT1)-lineage pituitary neuroendocrine tumors are composed of PIT1-lineage cells with cytological atypia and limited differentiation. These tumors are rare and no cytological features of this neoplasm have been reported. This study is the first to report the cytological features of an immature PIT1-lineage tumor. A 15-year-old boy with mild and asymptomatic hyperprolactinemia underwent endoscopic endonasal transsphenoidal resection of a sellar tumor mass associated with areas of calcification. Intraoperative smear cytology revealed high cellularity and discohesive cells. Large, bizarre nuclei, nuclear irregularities, and pseudonuclear inclusions were present. Histological examination also revealed large nuclei and nuclear irregularities. Immunohistochemically, the tumor cells were diffusely positive for PIT1 and focally positive for thyroid-stimulating hormone, growth hormone, and prolactin. Ultrastructural examination of the tumor specimen revealed large cells with pseudonuclear inclusions and nuclear irregularities. These findings are consistent with those of immature PIT1-lineage tumors. Smear cytology findings, including large cells, pseudonuclear inclusion bodies, prominent nucleoli, epithelial clusters, and mitotic activity, are useful for the rapid intraoperative diagnosis of immature PIT1-lineage tumors.

## Introduction

The pituitary gland has two major components: the adenohypophysis and the neurohypophysis. The tumors of adenohypophysial hormone-secreting cells have been traditionally classified as pituitary adenomas. However, they are now called pituitary neuroendocrine tumors (PitNETs) in the WHO's 5th edition because adenohypophysial cells are members of the neuroendocrine cell family [1,2]. PitNETs are now classified based on cell lineage as determined by the expression of transcription factors, hormones, and other biomarkers [1,2]. Immature PIT1-lineage PitNETs comprise PIT1-lineage cells with cytologic atypia and limited differentiation into thyrotrophs, somatotrophs, and/or lactotroph cells [1,2]. The majority of these tumors are clinically non-functioning, thus explaining the prior taxonomy of “silent subtype 3 pituitary adenomas" [3-5]. This invasive PitNET subtype is usually known for its aggressive biology and proclivity for tumor recurrence [3-5]. Because these tumors are rare, there are currently no cytological descriptions. This study is the first to document the cytological findings of immature PitNETs and discuss their differential diagnoses.

## Case presentation

**Figure 1:**
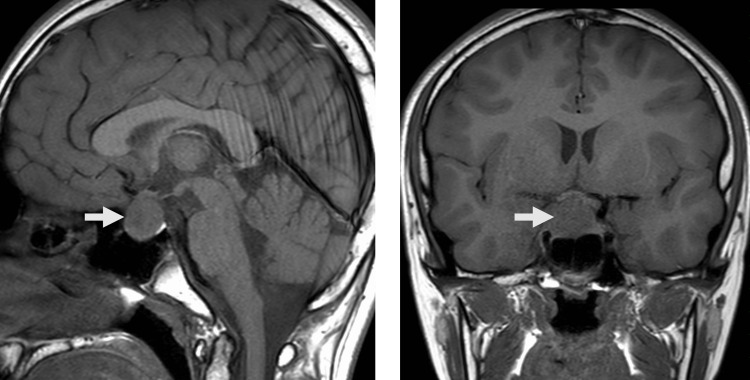
Magnetic resonance imaging (MRI) of the head MRI showing a sellar and supracelluar mass with axial T1-weighted MRI (A) and coronal T1-weighted MRI (B).

A 15-year-old boy first presented with transient, disabling headaches associated with nausea and vomiting two years ago. Magnetic resonance imaging (MRI) of the head at the hospital during the initial presentation revealed a homogeneously enhanced intrasellar and suprasellar tumor mass with areas of calcification measuring 20 mm in the craniocaudal direction. The patient was monitored during follow-up because there were no other symptoms related to the lesion. The frequency and intensity of headache attacks gradually increased over the next two years. A follow-up MRI confirmed that the sellar tumor had grown to 23 mm in the craniocaudal dimension with chiasmal compression (Figure 1).

An ophthalmological examination confirmed the absence of abnormalities. Laboratory studies, including hormone-loading tests for luteinizing hormone-releasing hormone, growth hormone (GH)-releasing peptide 2, and corticotropin-releasing hormone, did not reveal any dysfunction of the pituitary axis. The patient's concurrent mild hyperprolactinemia could be explained by the pituitary stalk effect (Table 1).

**Table 1:** Baseline anterior pituitary function tests before and after treatment ACTH, adrenocorticotropic hormone; FSH, follicle-stimulating hormone; FT4, free thyroxine; GH, growth hormone; IGF-1, insulin-like growth factor-1; LH, luteinizing hormone; PRL, prolactin; TSH, thyroid-stimulating hormone.

Tests	Results		Reference range
	Pretreatment	Posttreatment	
Free cortisol, μg/dL	8.4	3.5	3.7–19.4
ACTH, pg/mL	36.5	22.1	7.2–63.3
FT4, ng/dL	1.39	1.18	0.7–1.48
TSH, mIU/L	1.39	1.35	0.35–4.94
IGF-1, ng/mL	333	-	141–552
GH, ng/mL	0.92	-	<2.47
FSH, mIU/mL	3.6	-	2–8.3
LH, mIU/mL	2.4	-	1.2–7.1
PRL, ng/mL	38.8	4.8	3.5–19.4
Free testosterone, pg/mL	2.91	-	4.6–16.9

A binostril endoscopic endonasal transsphenoidal resection was performed because of the enlargement of the pituitary tumor. The firm, non-invasive tumors present were completely removed using the pseudocapsule-based extracapsular resection technique and sent for histological evaluation. The patient’s postoperative course was uneventful and the disabling headaches disappeared. His serum prolactin levels also normalized. A follow-up MRI performed 12 months after surgery revealed no evidence of tumor recurrence.

Cytological and histopathological findings

**Figure 2:**
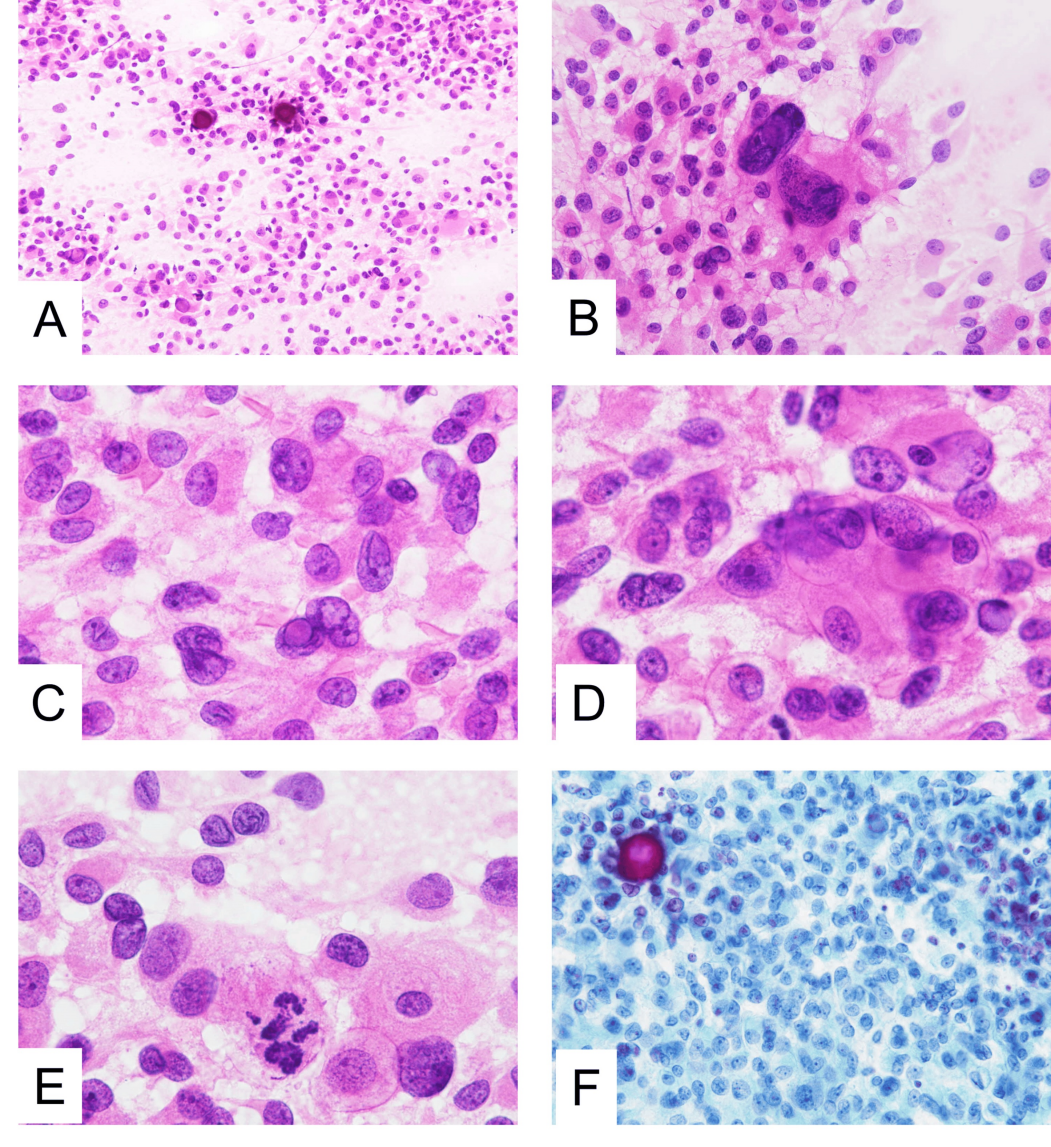
Cytological findings of a PIT1-lineage pituitary neuroendocrine tumor PIT1, Pituitary-specific transcription factor 1; H&E, hematoxylin and eosin. A: Squash smear showed high cellularity and various-sized nuclei. Calcification was occasionally seen (H&E staining, ×20). B: A touch smear showed large, irregular-shaped nuclei. Binuclear cells were also present (H&E staining, ×40). C: A pseudonuclear inclusion (*) and nuclear irregularity were visible (H&E staining, ×100). D: Epithelioid tumor cell clusters were noted (H&E staining, ×100). E: Mitosis was seen (arrow) (H&E staining, ×100). F: A pseudonuclear inclusion and nuclear irregularity were present (Pap staining, x40)

Tissue samples from the seller mass resected in this study were sent for intraoperative examination to the Department of Diagnostic Pathology at the University of Miyazaki Hospital. The tissue samples were smeared between two glass slides, immediately fixed with 95% ethanol, and stained with hematoxylin and eosin. After the operation, we made a Papanicolaou-stained smear. Intraoperative squash smear cytology revealed high cellularity and dyscohesive tumor cells (Figure 2A). Calcification was occasionally observed. These tumor cells had acidophilic or eosinophilic cytoplasm and also nuclei of varying sizes. Touch smears showed large irregularly shaped nuclei (Figure 2B). Pseudonuclear inclusions and prominent nucleoli were also often observed (Figure 2C). Focal epithelioid clusters and mitosis were also observed (Figures 2D, 2E). The patient was intraoperatively diagnosed with pituitary adenoma/PitNET and nuclear atypia. Papanicolaou-stained smears showed nuclear irregularities and pseudonuclear inclusions (Figure 2F).

**Figure 3:**
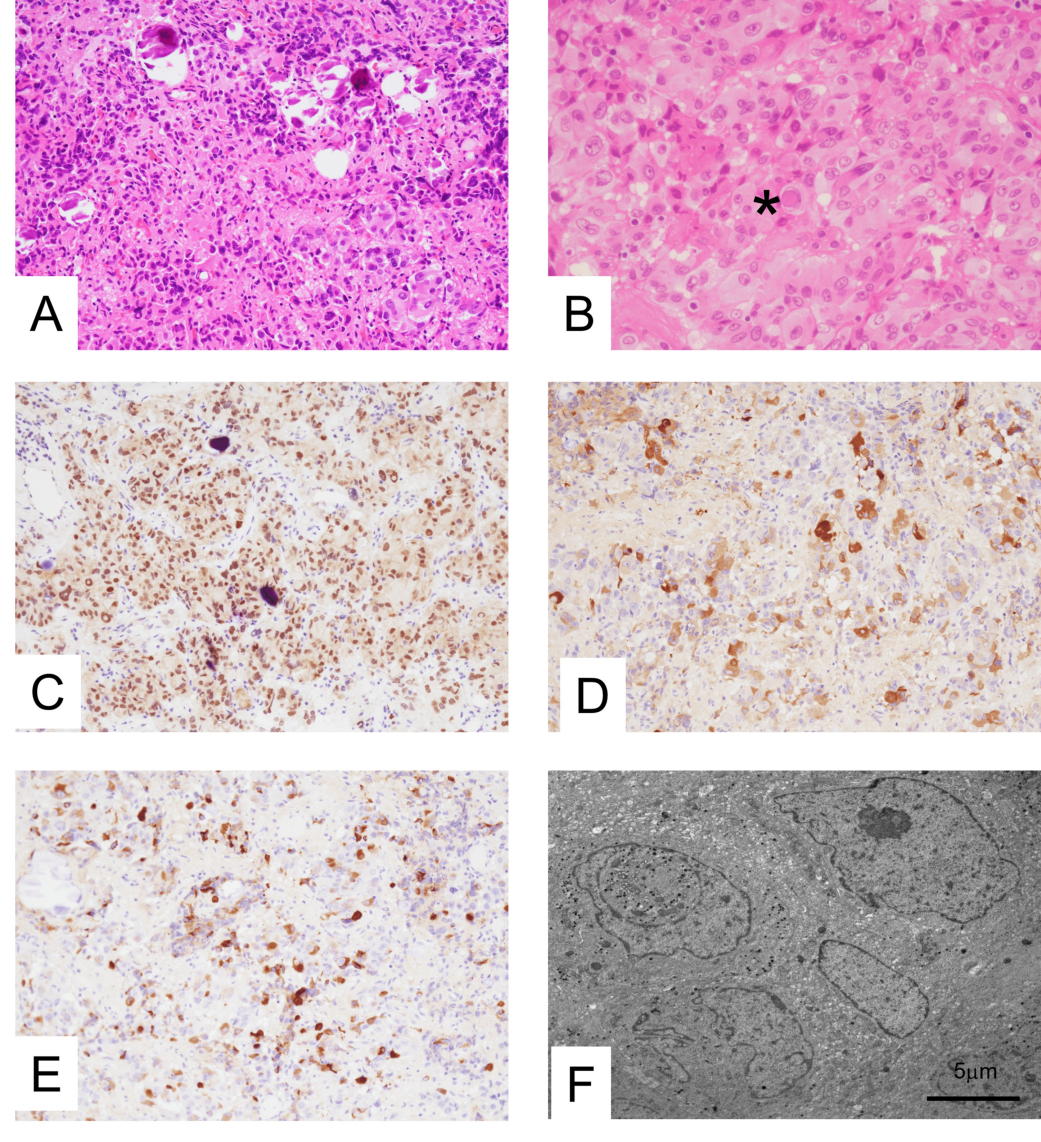
Histological, immunohistochemical, and ultrastructural findings of a PIT1-lineage pituitary neuroendocrine tumor PIT1, pituitary-specific transcription factor 1; H&E, hematoxylin and eosin; TSH, thyroid-stimulating hormone; GH, growth hormone. A: Histologically, tumor cells showed diffuse proliferation of polygonal cells. Calcification was occasionally seen (H&E staining, ×20). B: Tumor cells had an acidophilic cytoplasm and various-sized nuclei. A pseudonuclear inclusion (*) was present (H&E staining, ×40). C: Tumor cells were diffuse positive for PIT1 (×20). D: Tumor cells were patchy positive for TSH (×20). E: Tumor cells were focally positive for GH (×20). F: Ultrastructural findings of the tumor showed a pseudonuclear inclusion and nuclear irregularity (bar, 5 μm).

Histological examination of the tumor specimens revealed a diffuse or nodular proliferation of polygonal-to-spindle tumor cells. Calcification and fibrous stroma were also observed (Figure 3A). The tumor cells had an acidophilic cytoplasm and nuclei of varying sizes. Pseudonuclear inclusions, macronucleoli, and nuclear irregularities were also occasionally observed (Figure 3B). The mitoses were scattered. Immunohistochemistry revealed that these tumor cells were diffusely positive for PIT1 (Sigma Life Science, St Louis, MO, USA) (Figure 3C) and focally positive for thyroid-stimulating hormone (TSH; Dako, Glostrup, Denmark) (Figure 3D), GH (Dako, Glostrup, Denmark) (Figure 3E), prolactin (PRL; Dako, Glostrup, Denmark), and estrogen receptor (Novocastra, Leica Biosystems Newcastle Ltd, New Castle, UK). The antigen Ki-67 (Dako, Glostrup, Denmark) positivity rate was 6%. These tumor cells were perinuclear and strongly positive for cytokeratin (CAM5.2; Becton, Dickinson and Company, Franklin Lakes, NJ, USA) and GATA binding protein 3 (GATA3; Nichirei Biosciences Inc., Tokyo, Japan) but negative for T-box transcription factor (T-PIT; Sigma Life Science, St Louis, MO, USA) or steroidogenic factor 1 (SF1; Cosmo Bio Co. Ltd., Tokyo, Japan).

Ultrastructural examination of the tumor specimen revealed large cells with pseudonuclear inclusions and nuclear irregularities (Figure 3F). Small secretory granules were scattered along the cell membrane.

## Discussion

The current WHO classification of PitNETs or pituitary adenomas is based on the expression of pituitary-related transcription factors such as PIT1, SF-1, TPIT, and anterior pituitary hormones [1,2]. PIT1-lineage tumors include somatotroph (GH), mammosomatotroph (GH+PRL), lactotroph (PRL), thyrotroph (TSH), mature plurihormonal PIT1-lineage, immature PIT1-lineage, acidophil stem cells, and mixed somatotroph-lactotroph PitNETs. Immature PIT1-lineage PitNETs are defined as PIT1-lineage tumors with cytologic atypia and limited differentiation towards thyrotroph, somatotroph, and/or lactotroph cells [1,2]. In the present case, the tumor showed proliferation of the anterior pituitary tumor cells with nuclear atypia. Immunohistochemically, the tumor cells were diffusely positive for PIT1 and also focally positive for TSH, GH, and PRL. Therefore, we diagnosed the patient with an immature PIT1-lineage PitNET/pituitary adenoma.

The histological features of immature PIT1-lineage PitNETs have been reported [3-6]; however, their cytological findings have not been described. The cytology of the present case showed nuclei of various sizes and pleomorphisms. Pseudonuclear inclusions, macronucleoli, and nuclear irregularities were also occasionally observed. In addition, mitoses were present. In previous studies, the cytological findings of PitNETs and pituitary adenomas have been described as monolayered discohesive cells [7-9]. In contrast to our case, nuclear atypia was usually mild, and mitosis was not observed. Pseudonuclear inclusions, macronucleoli, and nuclear irregularities were also observed in the histological specimens studied. We believe that these nuclear findings are characteristic features of immature PIT1-lineage PitNETs.

Cytologically, immature PIT1-lineage PitNETs mimic malignant lymphomas, plasma cell tumors, and metastatic adenocarcinomas. Malignant lymphomas or plasma cell tumors consist of a sheet of monomorphic round tumor cells that may have prominent nucleoli but no bizarre large nuclei, moderate acidophilic cytoplasm, and epithelial clusters [10]. In addition, these tumors are uncommon among seller’s lesions. Metastatic adenocarcinomas typically exhibit more cohesive clusters with glandular or papillary structures [11]. Immature PIT1-lineage tumors overlap with their mature counterparts. Mature PIT1-lineage tumors have more cytological uniformity, with abundant acidophilic cytoplasm and extensive hormone immunoreactivities. They also generally lack nuclear atypia, macronucleoli, and nuclear pseudoinclusions [1,2]. Other lesions of the sellar region include craniopharyngiomas and Rathke's cleft cysts. Craniopharyngiomas often show clusters of squamous epithelium and ‘wet keratin’ (keratinized squamous cells with a ghost outline of dead nuclei). Rathke’s cleft cysts are lined with cuboidal to ciliated epithelia.

Immature PIT1-lineage tumors are rare, accounting for roughly one to three percent of all PitNETs [1-5]. In three large studies, the male-to-female ratio was close to 1:1 and the average age at presentation was approximately 40 years. Our patient was younger than the mean age of patients with immature PIT1-lineage PitNETs. Radiological examination showed that macroadenomas and the majority of immature PIT1-lineage tumors are clinically non-functioning, thus explaining the prior taxonomy of “silent subtype 3.” The present case involved a non-functioning macroadenoma and the preoperative hormonal evaluation was normal.

The distinction between immature PIT1-lineage tumors and other pituitary adenomas is of clinical significance, given their aggressive behavior. Previous studies have reported about 30-50% recurrence in immature PIT1-lineage tumors [3-5]. Several reports have documented that high proliferation rate and invasive growth are associated with tumor recurrence [12,13]. The present case had a high Ki-67 percentage, but no invasive growth, and the tumor tissue was completely resected. Although there was no evidence of tumor recurrence on radiographic examination, follow-up examinations may be necessary.

## Conclusions

This case report highlighted a rare case of immature PitNET. This patient had symptoms, and an MRI revealed a sellar tumor. This is the first report of the cytological findings in immature PIT1-lineage tumors. Touch smear cytology of typical pituitary adenomas shows monotonous, loosely cohesive plasmacytoid tumor cells. Cytology of this case demonstrated bizarre large cells, pseudonuclear inclusions, prominent nucleoli, and epithelial clusters. Cytology findings showed these atypical features, and histology, immunohistochemistry, and ultrastructural study confirmed immature PitNET. These findings are useful for the rapid intraoperative diagnosis of immature PIT1-lineage tumors in the future.
